# Overall scores as an alternative to global ratings in patient experience surveys; a comparison of four methods

**DOI:** 10.1186/1472-6963-13-479

**Published:** 2013-11-19

**Authors:** Maarten W Krol, Dolf de Boer, Jany JDJM Rademakers, Diana M Delnoij

**Affiliations:** 1 Netherlands Institute for Health Services Research (NIVEL), P.O. Box 1568, 3500 BN Utrecht, The Netherlands; 2 Institute for Quality in Health Care, Diemen, The Netherlands / TRANZO (Tilburg University), Tilburg, The Netherlands

**Keywords:** Quality of care, Composite measures, Nursing home, Consumer information

## Abstract

**Background:**

Global ratings of healthcare by patients are a popular way of summarizing patients’ experiences. Summary scores can be used for comparing healthcare provider performance and provider rankings. As an alternative, overall scores from actual patient experiences can be constructed as summary scores. This paper addresses the statistical and practical characteristics of overall scores as an alternative to a global rating in summarizing patient survey results.

**Methods:**

Data from a 2010 patient experience survey for approximately 12,000 nursing home residents (7.5% of all Dutch nursing home residents at the time) from 464 nursing homes in the Netherlands (25% of the Dutch nursing homes) was used. Data was collected through specifically designed standardized interview surveys. The respondents’ scores for 15 established quality indicators (or composites) for nursing home care were used to calculate overall scores for each nursing home, using four different strategies. The characteristics of the overall scores were compared against each other and with the respondents’ global rating.

**Results:**

The individual indicators showed stronger associations with each of the four overall strategies than with the global ratings. Furthermore, the dispersion of the overall scores across nursing homes was greater. Differences between overall scores appeared limited.

**Conclusions:**

Overall scores proved more valid than global ratings as a summary of the indicator scores, and also showed more pronounced differences between nursing homes. Because of the limited statistical differences between the strategies, and for practical reasons, a straightforward averaging of quality indicator scores may be preferred as an overall score.

## Background

For the past two decades, use of patient experience surveys as measurements of healthcare quality has increased substantially [1,2]. The results of these measurements may be used for various purposes by different stakeholders. For instance, patient experiences may enable healthcare providers to identify care elements or processes that their patients find unsatisfactory [3,4]. If patient surveys are standardized, the responses can be used to compare the quality of care delivered by different providers [5]. Patients can use this information to decide which healthcare provider they will use [1,6]. This information can also be used by healthcare regulators or inspectorates to assess the overall quality of healthcare, by researchers for studying healthcare systems, or for rewarding good quality of care [7].

Patient experience surveys usually include questions about a wide variety of healthcare characteristics, such as accessibility of healthcare, contact with healthcare providers and treatment information. Using commonly accepted methods of data reduction such as factor analysis and reliability analysis, the survey items are grouped to represent quality indicators, resulting in a quality rating for each indicator (also known as composites) [8,9]. Examples of quality indicators are the attitude of providers, perceived competence of providers or the information received about treatments or medication.

However, stakeholders often still feel they are presented with a wide variety of quality ratings, without a clear overall view of the results [10-13].

In many surveys, patients are asked to rate the overall quality of the healthcare provider, usually called a ‘global rating’. Although there are examples of global ratings in other settings, the most commonly used global rating in patient surveys consist of a single question: “How would you rate the health care provider?”, involving a scale from 0 to 10. Global ratings are often used as a summary measure [9,14]. However, it is questionable whether a single rating is a valid representation of the entire range of experiences reported in a patient survey. Research has shown that the global rating largely represents patients’ experiences with the process of care (e.g. communication), even though patients also consider many other aspects of care to be highly relevant [9,14,15]. Thus, there is a substantial risk that a global rating represents only some of the patient experience indicators.

As an alternative, overall scores may be considered as summary scores of quality of care. Overall scores can be constructed retrospectively from all quality indicators of a patient survey that are considered relevant. This should ensure that all indicators are represented by the overall score and accordingly, such an overall score may constitute a more valid summary score compared to the global rating.

The possibility of constructing overall scores has been explored for quality scores based on patient or hospital records [16,17]. Although we have heard of overall scores being used in patient experience research, there is limited peer reviewed evidence on their statistical properties, as far as we are aware. It is therefore useful to study to what extent such overall scores are indeed a better representation of the various aspects of patient experiences in healthcare than global ratings. In doing so, however, some methodological challenges arise. For instance, should all quality indicators be considered equal or should weighting factors be considered? And if so, what are the consequences of using different weighting factors?

The present study explores the possibility of constructing overall scores from a variety of quality indicators based on patient experiences, and addresses the following research questions:

Are individual indicator scores better reflected by overall scores than by global ratings? (Validity)

Do the overall scores vary between providers? (Discriminatory power)

Are overall scores to be preferred over global ratings and if so, which method is most suitable?

## Methods

### Data collection

Data was used from the Consumer Quality (CQ) index for nursing home care [18]. The CQ-index is a family of surveys, specific for one disease or provider, that are used in the Netherlands to measure and report patient experiences with healthcare [2,19]. The data for the CQ-index for nursing home care was gathered through structured interviews with residents of nursing homes (or homes for the elderly), conducted by qualified interviewers. This survey was constructed from topics deemed relevant by all stakeholders involved (e.g. clients, branch representatives, health insurance companies). After initial psychometric testing, quality indicators were identified that each consisted of one or more survey questions. Data from this survey was selected for the purpose of the present study as it is a very rich dataset, both in sample size and in the number of validated quality indicators (15 in total), each covering a specific element of the healthcare process [4,18]. Eleven of these indicators are constructed from two or more items (Cronbach’s alpha 0.61–0.81) and four consist of a single item. Where quality indicators consisted of more than one survey item, indicator scores were constructed by calculating the average over the items for each respondent, provided that the respondent answered half or more of the items for that indicator.

The original dataset used in this article consisted of 12,281 patient surveys, constituting 7,5% of all Dutch nursing home residents at the time. The surveys came from 464 nursing homes, about 25% of the Dutch nursing homes [20]. Since all Dutch nursing homes are legally required to participate in CQI research once every two years, bias in the selection of nursing homes in the present study is highly unlikely. Survey data was gathered through interviews with nursing home residents, conducted in the first half of 2010. Unfortunately, no information was available about the non-respondents. However, in the current setting, non-response on the CQ-index nursing home care has never been a problem [18].

### Data selection

Indicator scores ranged from 1 to 4. Respondents were only included in the calculation of the overall scores if they had given scores for at least 12 of the 15 indicators. 11,451 of 12,281 respondents met this condition and were eligible for our analyses (93%). The respondents’ characteristics are presented in Table 1. The number of respondents per nursing home varied between 8 and 82, with an average of 25 respondents (SD 6). The age of respondents ranged from 18 to 108. However, 98% of respondents were 60 years of age or older, with an average of 84 years.

**Table 1:** Respondent characteristics

		**N**	**Mean (SD)**
**Age (years)**		11,451	84.5 (8.5)
		**N**	**%**
**Education**			
	No education or primary education only	6,129	53.5
	Lower secondary education (*reference*)	3,620	31.6
	Higher secondary education or higher	1,702	14.9
**Self-reported health**			
	Good	5,030	43.9
	Moderate (*reference*)	5,183	45.3
	Poor	1,238	10.8
**Years of residence**			
	Less than 1 year	2,696	23.5
	Between 1 and 2 years	2,476	21.6
	Between 2 and 5 years (*reference*)	3,499	30.6
	More than 5 years	2,780	24.3
Gender*			
	Male	2,984	26.1
	Female	8,439	73.9

*: not used as case mix adjuster.

### Overall score construction

We examined four possible strategies for constructing overall scores. Each of those strategies is presented in detail in this section.

For the **Average Overall Score**, the indicator scores for each respondent were averaged (arithmetic mean), as individual overall scores. The average overall score over all its residents provided the overall score for each nursing home. This is the most straightforward way to construct an overall score for a provider.

The **Patient Perspective Overall Score** was calculated by adjusting each indicator score for the importance that patients attribute to the specific quality indicator. These importance scores were measured during the development of the survey by asking respondents to rate the importance of each survey item on a scale from 1 (not at all important) to 4 (very important) [18]. The importance of each indicator was calculated as the mean importance of the underlying items. For instance, the three items on indicator 1.1 (bodily care) had an average importance of 2.97, whereas the mean importance over all 15 indicators was 3.10. This means that bodily care is of less than average importance for nursing home residents. For each respondent, indicator scores were adjusted for their relative importance. So for indicator 1.1, indicator scores were given a weighting of 0.96 (=2.97/3.10), thereby decreasing their contribution to the overall score. Conversely, scores on indicators with higher than average importance were given a higher weighting. Doing this means that the indicators that are important to respondents are emphasized. After these adjustments, the indicator scores were averaged for each respondent. Subsequently, the average of the residents’ overall scores provided the overall score for each nursing home.

The third strategy, the **Differences Overall Score**, took account of differences between providers in indicator scores. By adjusting quality indicators for their variance, differences between providers in indicator scores may be expanded. One way of doing this is to calculate the intraclass correlations (ICC), which show the variation in indicator scores that can be attributed to differences between providers [21,22]. To obtain the ICC, multilevel analyses were performed for each of the indicators (empty 2-level models). Coming back to the example of indicator 1.1 (Bodily care), the analysis showed that its ICC was 0.11. This meant that 11% of the variation in scores on this indicator could be attributed to differences between nursing homes. However, the mean ICC over all 15 indicators proved to be 0.15. In other words, scores on indicator 1.1 showed less differentiation between nursing homes than the average across all indicators. Indicator scores were then adjusted according to their relative ICC. In the case of indicator 1.1, individual scores were given a weighting of 0.73 (=0.11/0.15), thus decreasing their contribution to the overall score. Conversely, scores on indicators with a higher than average ICC were given a higher weighting. Differences between providers are thus emphasized; indicators on which there is relatively more differentiation are weighted more heavily in the overall score than indicators with little differentiation. After this adjustment, the indicator scores were averaged for each respondent. Subsequently, the average of the residents’ overall scores provided the overall score for each nursing home.

Finally, the fourth strategy (**Average Rating Overall Score**) involved a ‘star rating’ for each of the individual indicator scores. These stars are awarded based on the dispersion of scores on each indicator and subsequently on the statistical differences between the providers: two stars for an average performance, one for the worst performers and three for the best performers. Providers with three stars perform significantly better on an indicator than providers with one star [23]. These stars are a standard part of provider feedback reports on CQ-index survey results, enabling providers to compare their performance against that of others.

The overall score was constructed by averaging the number of stars per provider over all quality indicators. This overall score can only be constructed using aggregated data, as each individual indicator score depends on the scores of all other providers, as described in the *Data Analyses.*

The **Global Rating** of quality consisted of a single question: “How would you rate the nursing home?”. It involved 11 response categories, ranging from 0 to 10, in which ‘0’ was labelled ‘the worst possible nursing home’ and ‘10’ was labelled ‘the best possible nursing home’. The residents’ ratings were averaged for each nursing home.

### Data analyses

The individual indicator scores and the individual overall scores were both used in multilevel analyses [24]. Scores per nursing home were adjusted for differences in case mix between homes, using the commonly accepted case mix variables of age, educational level and self-reported health of the respondent [23,25]. In addition, an adjustment was made for the length of stay [18]. Empirical Bayes Estimation (EBE) was used to estimate case mix-adjusted means per nursing home for each of the quality indicators and overall scores [24,26-29].

The Average Rating Overall score can only be calculated after the multilevel analyses. Based on confidence intervals, organizations receive either one, two or three stars for each quality indicator. Therefore, the average number of stars over all quality indicators can already be seen as an overall score in itself. The Average Rating Overall Score, however, is difficult to compare with the other three overall scores. Its approach is totally different and so is its scale (1 to 3 versus 1 to 4). Also, a number of statistical properties of this composite cannot be analysed: it is not possible to calculate an intraclass correlation or its reliability.

To answer our first research question, Pearson correlation coefficients were calculated between individual indicators and the overall scores (and global rating) to assess the validity of the latter. The greater the association between individual indicators and a composite, the better that overall score reflects individual indicator scores. Fisher’s z-transformation was used for averaging correlation coefficients [30]. Interpreting a correlation coefficient is highly dependent of the context in which it is calculated. In the case of patient experience research, correlation coefficients between survey items are considered high when 0.7 or above, while 0.4 and lower is considered a weak relationship [31]. With regard to our second research question (assessing discriminatory power), intraclass correlations (ICC) were calculated from the multilevel analyses. As with the Pearson correlations, there is no gold standard with regard to cut-off points for the ICC. The higher the ICC, the more the variance in scores can be attributed to the nursing home a respondent is living in. Thus, a higher ICC is preferable in view of discerning between provider performances. Differences in rankings of providers were also calculated in order to assess the influence of each of the overall score constructs and the global rating on the position of providers. In this regard, the influence of sample size will also be considered. Our third research question will be answered by assessing the results of the two other research questions, combined with the practical applicability of the four strategies.

Analyses were performed using STATA 11.0 (StataCorp. 2009. *Stata Statistical Software: Release 11*. College Station, TX: StataCorp LP.).

## Results

### Overall score characteristics

The first three overall scores prove to be equally reliable scales at the level of individual respondents (Cronbach’s alpha 0.80-0.81, data not shown). Also, the Average, Patient Perspective and Differences Overall Scores are quite similar in terms of the results at the provider level, as can be seen from Table 2; the ranges of the means and standard deviations are only 0.060 and 0.008 respectively.

**Table 2:** Characteristics of overall scores at the provider level

**Composite**	**Mean**	**SD**	**Min**	**Max**	**ICC**	**Reliability ICC**	**Required N (rel. = 0.80)**
Average	3.359	0.164	2.820	3.709	0.229	0.87	13.5
Patient perspective	3.350	0.163	2.822	3.708	0.226	0.87	13.7
Differences	3.410	0.171	2.774	3.750	0.282	0.90	10.2
Average rating	2.051	0.397	1.067	2.933	NA	NA	NA
Global rating	7.640	0.260	6.752	8.400	0.076	0.65	48.9
N (organizations): 464							

From additional analyses (data not shown), it is clear for the Patient Perspective that the effect of weighting indicator scores by their importance is limited: the largest adjustment was made on indicator 6.1 (Care plan), for which the scores were given a weighting of 0.80. The other indicator adjustments are between 0.90 and 1.12. As a result, it yields similar results to the Average Overall Score. For the Differences Overall Score, however, the adjustments are more substantial. The largest adjustment in scores is on indicator 2.3 (Housing and privacy): this was given a weighting of 3.00. The other indicator adjustments are between 0.35 and 1.45. Also, the adjustments for the Patient Perspective and Differences Overall Score go opposite ways for a number of indicators, but in the same direction for others.

Another important aspect is the sample size needed per nursing home if reliable discrimination between them is to be possible based on their performance ratings. The required sample sizes for the overall scores prove to be quite small, as shown in the last column of Table 2. This is due to the relatively large differences in overall scores between organizations. The required sample sizes for the overall scores also proved to be smaller than for the global rating.

### Reflection of the quality indicators (Validity)

The validity was tested by examining how the individual quality indicator scores were reflected in the overall scores. For this purpose, correlations of the individual quality indicators against the overall scores were calculated. The results are shown in Table 3. The individual indicators differ in the extent to which they are reflected in the overall scores: some indicators are more related to the overall scores than others. Seven of the indicators have a strong relationship with all individual overall scores (correlation >0.7). There are limited relationships (correlation <0.4) for two indicators: arrangements between the resident and the nursing home (6.1) and the quality of cleaning (2.1). On average, however, the overall scores are substantially correlated with the individual indicator scores: 0.67 to 0.69 (using Fisher’s z-transformation) [30]. The strengths of the correlations are broadly similar between the different overall scores.

**Table 3:** Correlations between indicator scores, overall scores, and global rating

**Indicator**	**Average**	**Patient perspective**	**Differences**	**Average rating**	**Global rating**
1.1 Bodily care	**0.79**	**0.79**	**0.77**	**0.77**	0.56
1.2 Meals	0.57	0.58	0.50	0.53	0.53
2.1 Comfort	0.46	0.47	0.38	0.42	0.37
2.2 Atmosphere	**0.79**	**0.79**	**0.76**	**0.76**	0.54
2.3 Housing and privacy	0.55	0.54	0.68	0.52	0.28
2.4 Safety of living environment	0.60	0.60	0.56	0.58	0.40
3.1 Activities	0.61	0.60	0.58	0.59	0.37
3.2 Autonomy	0.57	0.55	0.66	0.57	0.30
4.1 Mental well-being	**0.79**	**0.79**	**0.76**	**0.78**	0.61
5.1 Competence and safety of care	**0.83**	**0.84**	**0.80**	**0.82**	0.57
5.2 Attitude and courtesy of care providers	**0.84**	**0.84**	**0.80**	**0.84**	0.62
6.1 Care planning and evaluation	0.35	0.33	0.37	0.36	0.16
6.2 Shared decision making	**0.75**	**0.75**	**0.73**	**0.74**	0.53
6.3 Information	0.64	0.63	0.59	0.60	0.39
6.6 Availability of personnel	**0.81**	**0.82**	**0.78**	**0.80**	0.61
Average correlation (Fisher’s z)	0.69	0.69	0.67	0.67	0.47
Global rating	0.68	0.69	0.64	0.66	NA
N = 464					

All correlations are significant at p < 0.001. Strong correlations (>0.7) in bold.

Individual indicators are more strongly associated with each of the overall scores, than they are with the global rating. All of the correlations between each of the four overall scores and the global rating are close to 0.7.

### Differentiation between providers (Discriminatory power)

The discriminatory power of the overall scores was tested by calculating the proportion of variance that is attributable to providers, i.e. in this case to the nursing home. This proportion is expressed in the intraclass correlation (ICC).

For the individual indicators, intraclass correlations (ICC) ranged from approximately 0.03 (Safety) to 0.40 (Housing and privacy) (data not shown). These ICC values are substantial, compared to analyses of other CQ-index data, which gave values up to 0.05 [32-35]. In other words, a large part of the variance in overall scores can be attributed to the nursing home. Moving back to Table 2, the ICCs for the four overall scores were between 0.22 and 0.28. Importantly, the ICC of each overall score is far higher than the ICC of the global rating (0.08). As expected, the Differences Overall Score shows the largest ICC, as we expanded differences in indicator scores between organizations.

Because overall scores are used for comparing healthcare providers, merely inspecting the differences in their distributions of scores is not enough. It is also essential to know what each strategy does to the ranking of the providers, as some stakeholders use performance data for this purpose. Ranking correlations (Kendall’s Tau) and differences in ranking were therefore calculated for each of the four overall scores and for the global rating. Table 4 shows the associations between the rankings of providers for each of the overall scores and for the global rating.

**Table 4:** Associations between provider rankings for global rating and overall scores

	**Average**	**Patient perspective**	**Differences**	**Average rating**	**Global rating**
Average	1.00				
Patient perspective	0.98	1.00			
Differences	0.91	0.89	1.00		
Average rating	0.85	0.85	0.84	1.00	
Global rating	0.47	0.48	0.44	0.45	1.00
N (organizations): 464					

All correlations significant at p < 0.001.

From this analysis, it is clear that the global rating yields quite a different provider ranking than each of the overall scores; associations between this rating and the overall scores are low. The associations between each of the four overall scores, however, are considerable.

**Figure 1:**
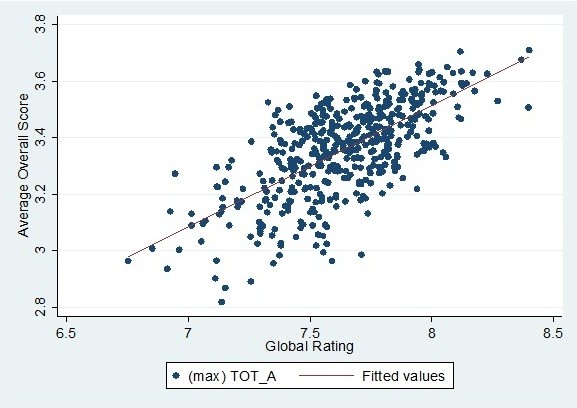
Scatterplot of the average overall score and global ratings (N = 464).

To assess the actual differences in ranking, they were calculated for each of the overall scores, using the global rating as a standard. Differences were expressed as the number of providers whose rank changed by more than 116 (25 per cent of the dataset) or even by more than 232 (50 per cent of the dataset). It turns out that for each of the overall scores, the rankings of an average of 145 providers (31%, range 139–149) would shift more than 116 places compared to the global rating. On average, 20 providers would even move by more than 232 places (4%, range 17–23). Differences between the global rating ranking and overall score rankings are therefore considerable, whereas differences in rankings between each of the overall scores are limited. It should be noted, though, that a large change in rankings does not necessarily reflect a large absolute difference in either overall scores or the global rating. Due to the clustering of the scores, a difference in ranking of 116 can be caused by an absolute difference as small as 0.09 on the Average Overall Score, for instance. For the global rating, the same applies for absolute differences as small as 0.16. To illustrate this, Figure 1 shows the relationship between the Average Overall Score and the global ratings of all providers, which is comparable for the three other overall score strategies. As can be seen from this figure, the scores of many providers are somewhat clustered. Nonetheless, the choice of the specific overall score strategy does have a severe impact on the rankings of several providers, especially the providers further removed from the reference line.

## Discussion and conclusion

In this study, four different strategies for constructing overall scores were assessed, and their characteristics compared to a global rating of quality of care.

With regard to our first research question, correlations between individual quality indicators and each of the overall scores proved to be considerable, in contrast to their rather weak associations with the global rating. This means that the specific patient experiences are better reflected by the overall scores than by a global rating. Overall scores therefore turn out to be a more valid way of summarizing the survey data than a global rating. It should however be noted that overall scores consist only of the scores actually reported by patients in the survey, whereas a global rating can be based on anything, including for instance on aspects of healthcare not mentioned in the survey. It is important to keep this in mind. For the association between overall scores and the global rating, correlations proved to be about 0.7. This is considerable, but nevertheless it is safe to state that a single question about the overall quality (i.e. global rating) does not necessarily produce the same result as an overall score calculated from validated quality indicators.

The overall scores showed considerable discriminatory power, even more so than the global rating. As a result, the overall scores enable more rigorous differentiation of providers, which is an important finding for future quality assessment of healthcare providers. In line with earlier research, the discriminatory power of the overall scores also decreases the number of responses required to obtain reliable scores, compared to individual indicators [36-38]. The same applies, although to a lesser extent, to the global rating.

We found profound differences between rankings based on the overall scores we constructed and the ranking based on the global rating. A large part of these seemingly substantial differences in ranking were due to clustering of scores, in which case a negligible difference in score may yield a huge difference in ranking. However, we also illustrated that for some of the providers, the global rating yielded a substantially different result compared to the overall scores, suggesting that for these providers it does matter whether they are classified based on a global rating or an overall score.

The effort required to construct meaningful overall scores as an alternative to global ratings does not seem to be in vain; their advantages over using a global rating are clear. But which strategy for constructing an overall score should be preferred? In the past, many stakeholders have suggested the use of the Average Rating Overall Score as a way of summarizing the performance of healthcare providers, because the star ratings per quality indicator are already in place for reporting on CQ-index data. Even though it shows promising results (Table 3), the nature of this overall score construct severely limits the requisite statistical analyses if it is to be compared with other overall scores. The other overall scores are constructed by calculating an average over all indicators *for each individual* which is then aggregated to a provider mean, i.e. these overall scores are an average of scores of individual respondents. In contrast, the average star rating is essentially an average of the *provider scores* for each indicator. We believe the latter strategy to be unfavourable, because conventional statistical parameters such as ICCs cannot be calculated. In addition, the interpretation of standard errors and confidence intervals will be different as these no longer depend on the number of individuals per provider, but on the number of indicators being measured.

When the three remaining strategies are compared, they seem to yield statistically similar results. The differences between providers are comparable (according to the calculated ICC’s) and there are similar and substantial correlations with the individual indicators. Choosing the ‘best’ strategy from these three overall scores does not seem to depend on either validity or discriminatory power and so may be allowed to be guided by practical considerations.

In this context, it is also valuable if the overall scores are easy to understand and to use for all stakeholders involved. The Average Overall Score strategy is the most straightforward to understand: it consists of merely averaging the scores of all the quality indicator scores. The other overall scores, however, require quite a distinct level of statistical literacy and need explanation. From this point of view, they are not to be preferred over the Average Overall Score. Therefore, the sound statistical basis plus above all the practical arguments make the simple Average Overall Score the best choice.

### Strengths and limitations

It is important to note that there is no ‘gold standard’ available for the measurement of patient experiences. Apart from the method used in our research, there are other possible ways of measuring a global rating. For instance using different wordings or a different scale. We cannot rule out the possibility that other methods concerning a global rating of care may lead to different outcomes. However, the way the global rating was measured in this research is the most commonly used strategy in patient surveys in the USA (CAHPS) and the Netherlands (CQ-index) [9,19].

Many stakeholders favour a global rating as a way of summarizing the patients’ opinions on health care, for its simplicity. However, patient experience surveys mean to cover all aspects of health care relevant to patients, health care providers and other stakeholders and it has been shown that not all of these aspects are represented by a global rating [14]. Since the present paper demonstrates that an overall score constructed from patient experiences represents the underlying health care aspects better than a global rating, an overall score seems at least as valid in summarizing patient experiences as the global rating, if not more.

We thoroughly investigated the properties of four possible overall score constructs using a large dataset containing patient experiences of a quarter of all Dutch nursing homes. As a result, our findings should be fairly representative for the Dutch setting of nursing homes. Also, our data contained a large number of quality indicators, allowing us to assess the validity of the overall scores on many different aspects of healthcare.

The construction of quality indicators does involve a risk regarding nonresponse, however. Structural nonresponse on items with a notably high or low average score may influence quality indicator scores. If nonresponse differs between institutions, it may lead to unjustified differences on that particular quality indicator. The same goes, to a lesser extent, for the construction of the overall scores; we allowed for a maximum of three missing quality indicator scores at patient level. However, our stringent approach with regard to missing values on quality indicators made selective missing values on the overall scores at provider level highly unlikely. If this would indeed be the case, less missing values per quality indicator or overall score should be allowed.

It is possible that the analysis of different survey data would yield different results. In other words, the specific properties of these overall score constructs have yet to be established for other patient surveys. Although differences between most of the constructs proved to be limited in our research, this may not be the case for other datasets, as is also shown in other studies [16,37,39,40]. Also, there are a number of strategies for calculating overall scores that we have not included in this research. Well-known examples are ‘all-or-none’ (providers score a ‘1’ if they meet a certain quality criterion and a ‘0’ if they do not, after which all quality scores are summed) and the ‘percentage of success’ (percentage of quality criteria met) [39,41]. But as the indicator scores from the current data can be considered as continuous variables, these and many other strategies were not applicable. However, we concede that there are other applicable construction methods that could have been considered for this study.

### Practical implications

Based on our results, we would recommend the use of an overall score as a more valid and reliable alternative to the global rating in summarizing patient survey results. However, a few practical issues should be considered in using overall scores.

Firstly, it is important to bear in mind that constructing overall scores will inevitably lead to a certain amount of data reduction, thus obscuring details and maybe even differences between organizations from the original data. Overall scores oversimplify results and are only useful for rough comparisons [37,42]. In our opinion, overall scores should not be presented as a substitute for individual indicator scores, but rather as a useful addition to survey results to provide a quick overview. For a more detailed picture, stakeholders may subsequently inspect the individual indicator scores; these show where specific differences between providers occur and which processes actually need improvement. This is also important in the case of individual indicators that do not seem to be reflected by an overall score.

Secondly, careless and uninformed use of (overall) scores may have serious consequences for healthcare organizations or individual healthcare providers, if used for quality ranking [16]. Finally, stakeholders may prefer one method of constructing overall scores over the others, based on their aims [16]. It is even possible to combine different constructs. Although this is theoretically interesting, such complex constructs will make it more difficult for stakeholders to understand and interpret the overall scores.

In the end, constructing overall scores remains a great challenge, which needs to be handled with care [16,36]. If the matters above are addressed, though, a well-defined overall score may present all stakeholders with a valid and reliable overall view of quality of care from the patients’ perspective.

## Competing interests

The authors declare that they have no competing interests.

## Authors’ contributions

MK participated in the design of the study, carried out the analyses and drafted the manuscript. DB designed the study, checked the analyses and co-authored the manuscript. JR was involved in the interpretation of the results and critical revision of the manuscript. DD was involved in the interpretation of the results and critical revision of the manuscript. All authors approved the final version of the manuscript.

## Pre-publication history

The pre-publication history for this paper can be accessed here:

http://www.biomedcentral.com/1472-6963/13/479/prepub
